# Recurring implementation determinants in digital health innovations: a multi-context multiple case study and cross-case synthesis into a four-domain analytical framework

**DOI:** 10.3389/fdgth.2026.1844330

**Published:** 2026-07-15

**Authors:** Mariusz Jaworski, Petra Petrová, Thomas Spittler, Radu-George Ciorap, Agnieszka Wojtczuk-Turek, Ilona Cieslak

**Affiliations:** 1 Department of Education and Research in Health Sciences, Faculty of Health Sciences, Medical University of Warsaw, Warsaw, Poland; 2 Department of Biomedical Technology, Faculty of Biomedical Engineering, Czech Technical University in Prague, Kladno, Czechia; 3 Deggendorf Institute of Technology, European Campus Rottal-Inn, Pfarrkirchen, Germany; 4 Grigore T. Popa University of Medicine and Pharmacy, Iași, Romania; 5 Institute of Human Capital, SGH Warsaw School of Economics, Warsaw, Poland

**Keywords:** digital health, health systems, healthcare implementation, implementation determinants, implementation science, multiple case study, organizational readiness, telehealth

## Abstract

Digital health technologies are increasingly used in healthcare, but their implementation in routine practice remains uneven. Many solutions are introduced as pilots but are not sustained, scaled up, or fully embedded in everyday organizational work. This study aimed to identify implementation determinants that recur across different real-world digital health settings and to organize them into a practical analytical framework for implementation planning and readiness assessment. We conducted a secondary qualitative multiple case study with cross-case synthesis. Thirteen digital health implementations from six European countries, developed within an Erasmus+ project, were analyzed using standardized implementation case reports. The cases covered a range of technologies, including telemonitoring, artificial intelligence, extended reality solutions, and digital platforms, implemented at different levels of healthcare systems. The reports described implementation context, technology type, stakeholders, barriers and facilitators, organizational conditions, and lessons learned. Because the material consisted of secondary standardized reports rather than primary qualitative data, the analysis focused on recurring reported determinants and did not aim to develop a fully inductive explanatory theory. Established implementation frameworks were used ex post to support interpretation. The analysis identified 20 implementation determinants. These were organized into four interdependent domains: technological, organizational, user-related, and system-level. Frequently reported issues included interoperability, usability, leadership and governance, workflow integration, staff competencies, user engagement, funding stability, and regulatory alignment. Across the cases, implementation challenges rarely concerned the technology alone. The resulting four-domain framework offers a concise way to structure implementation knowledge across heterogeneous digital health settings. It may support early identification of implementation risks, readiness assessment, and more systematic planning of digital health interventions. The framework is best understood as an analytical and planning tool, rather than as a model that predicts implementation success.

## 1 Introduction

Digitalization has become one of the major directions of change in contemporary health systems. International organizations, including the World Health Organization (WHO) and the Organization for Economic Co-operation and Development (OECD), point to the potential of digital technologies, such as telemedicine, remote monitoring, mobile applications, artificial intelligence (AI), interoperable data systems, and extended reality (XR) solutions, to improve access to care, quality of services, and system efficiency, particularly in the context of population ageing, workforce shortages, and the increasing burden of chronic diseases (1, 2). However, experience from real-world healthcare settings shows that the availability of technology is not sufficient to ensure successful implementation. Many digital health innovations remain limited to pilot projects, are not scaled up, or are discontinued once initial funding or project support ends (3, 4).

The implementation of digital technologies is shaped by more than technical performance alone. Whether a solution becomes part of routine practice depends on how well it fits existing organizational structures, whether patients and healthcare professionals are willing and able to use it, and whether the wider system context supports its adoption. Issues such as health policy, digital infrastructure, legal requirements, and financing models may therefore be as important as the technology itself (5, 6). This broader understanding of implementation is reflected in established frameworks, including the Non-adoption, Abandonment, Scale-up, Spread, and Sustainability (NASSS) framework (5) and the Consolidated Framework for Implementation Research (CFIR) (6), both of which help to organize the multiple factors involved in implementing health innovations.

Although these frameworks have been important for implementation research, much of the empirical literature on digital health still examines single technologies, individual programmes, or one clinical setting at a time. Such studies are useful, but they make it difficult to see which implementation issues appear across different technologies, organizations, and health systems. For this reason, comparative work across diverse digital health implementations is needed. It can help distinguish challenges that are highly context-specific from those that seem to recur across settings, such as problems of workflow integration, user acceptance, infrastructure, governance, or sustainability (7–9).

There is still limited evidence from studies that bring together several real-world digital health implementations and compare them using a common analytical approach. This is particularly relevant when the cases differ not only by technology type, but also by level of care, organizational setting, and national health system. Without such comparisons, implementation guidance often remains either very specific to one setting or too broad to guide concrete planning decisions. This creates a problem for teams preparing new digital health projects: they may know that implementation is complex, but have less support in identifying which risks should be anticipated across different types of technologies and care settings. More empirically grounded syntheses are therefore needed to translate implementation experience into tools that can be useful before and during implementation (10–12).

In response to this gap, this study analyzes real-world digital health implementations from different countries, healthcare settings, and levels of care. The included cases covered five broad types of technologies: (a) telehealth and remote monitoring solutions, such as video consultations and home-based monitoring for chronic disease management; (b) artificial intelligence-enabled diagnostic, screening, and decision-support systems; (c) extended reality (XR)-based therapeutic and rehabilitation interventions; (d) digital therapeutic, self-management, and patient-support platforms; and (e) assistive or socially interactive technologies used in therapeutic or care-related contexts.

This diversity was important for the analysis. Different digital health technologies do not create identical implementation problems. Telehealth and remote monitoring solutions often require changes in workflows, reimbursement arrangements, connectivity, and coordination between services (9, 13). Artificial intelligence-enabled tools may raise additional concerns related to data quality, explainability, regulation, and clinicians' trust (4). Extended reality and assistive technologies, in turn, may depend more strongly on usability, safety, equipment management, staff training, and user acceptance (14). For this reason, the cross-case synthesis did not treat digital health as a single uniform category. Instead, it examined which implementation determinants were reported across different technology types, while remaining attentive to technology-specific challenges.

A similar need for context-sensitive implementation analysis has been noted in other areas of digital transformation, including digital pathology, where successful adoption depends not only on technological performance but also on implementation strategy, organizational integration, and wider system-level conditions (15). More broadly, digital health implementation matters because digital tools can support access to care, resource use, and health system resilience, but only when they are embedded in routine practice in ways that fit local organizations and users (14, 16). At the same time, digital transformation may also reproduce or deepen existing inequalities if issues of infrastructure, digital literacy, affordability, and accessibility are not considered early in the implementation process (17, 18).

In this study, we use the identified determinants to organize what was reported across different digital health implementations. The aim is to identify recurring reported determinants and develop a four-domain analytical framework that can support readiness assessment and help identify possible bottlenecks across technological, organizational, user-related, and system-level areas.

## 2 Materials and methods

### 2.1 Study design

This study used a qualitative comparative design based on multiple case studies (19, 20). The aim was to identify determinants of digital technology implementation that were reported across different healthcare contexts. The multiple case study approach made it possible to examine real-world implementation experiences and compare patterns across cases (19). Each case was treated as a bounded account of an implementation process, rather than as an evaluation of a single technology.

This design was appropriate because digital health implementation is usually complex and context-dependent (5–8). It involves not only the technology itself, but also organizational structures, users, workflows, and system-level conditions. A multiple case study design allowed us to examine this complexity in natural implementation settings and to identify issues that appeared across different organizational and clinical contexts (19, 20).

The study had an exploratory–analytical character. It did not aim to test the effectiveness of a specific technology or compare clinical outcomes between solutions. Instead, it focused on reported implementation experiences, barriers, facilitators, and contextual conditions. The purpose was to identify recurring implementation determinants and organize them into an empirically informed analytical framework.

Methodologically, the study should therefore be understood as a multiple case study with cross-case synthesis. Each implementation case was defined by a specific digital technology, implementation setting, stakeholder configuration, and organizational context. The analysis compared cases not to rank them or assess their success, but to examine what types of implementation issues were reported across them. This approach is consistent with case study methodology, which is useful for studying complex implementation processes in real-world settings.

The reporting of implementation processes was partially aligned with the Guidelines and Checklist for the Reporting on Digital Health Implementations (iCHECK-DH) (21). The case reports included several elements recommended for describing real-world digital health implementations, such as the implementation context, characteristics of the technology and organization, stakeholder involvement, implementation barriers and facilitators, and reported lessons learned. Full alignment with iCHECK-DH was not possible because the analysis was retrospective and based on secondary case materials. As a result, some details, such as implementation timelines, formal implementation strategies, adaptation processes, standardized implementation outcome measures, and long-term sustainability plans, were not available for all cases. This limitation is consistent with broader concerns in the literature about the variable quality and standardization of digital health implementation reporting (22).

### 2.2 Data sources and case selection

The empirical material consisted of case studies describing real-world uses of digital technologies in healthcare. The cases covered several types of technologies, including telecare and telemonitoring, artificial intelligence-supported tools, digital patient platforms, extended reality (XR)-based interventions, and larger telemedicine implementations at regional or national level.

The case studies were prepared by partner institutions involved in the Erasmus+ project “Hybrid Healthcare—Advancing Hybrid Management and Digital Technology in Healthcare Education”. Six partner institutions contributed case materials, each representing a different healthcare system or organizational context. Within the project, each partner institution was asked to prepare four case studies on digital technology implementation. This gave an initial planned pool of 24 cases. The case descriptions were prepared between January and August 2025. To make the materials comparable, all case descriptions followed a standardized reporting template developed for the project. The template covered the implementation context, type of technology, course of implementation, stakeholders involved, organizational and clinical impact, reported barriers and facilitators, resources used, and key lessons learned.

Eleven cases were excluded because they did not provide sufficient evidence of real-world implementation. This included conceptual proposals, feasibility reports, educational or training tools, laboratory prototypes, and pilot activities without evidence of integration into clinical or organizational practice. Some cases were also excluded because the available information was too limited to confirm use by real users in a real-world healthcare or care-related setting. The lower final number of included cases reflected differences between partner institutions in their readiness to report complete implementation processes during the project period.

These inclusion criteria were used to keep the analysis focused on implementation processes occurring under real healthcare system conditions. This was consistent with the study aim and with implementation frameworks that emphasize the role of institutional context, organizational fit, and practical use in real-world settings (5, 6).

In total, 13 case studies met all eligibility criteria and were included in the analysis. The included cases varied by technology type, level of care, organizational context, and healthcare system. This allowed us to compare reported implementation determinants across a diverse set of institutional and clinical settings. An overview of the included case studies is presented in Table 1. Each case was assigned a unique identifier, from Case 1 to Case 13, which was used consistently throughout the analysis and reporting.

**Table 1:** Overview of included case studies organized by technology class.

Technology class	Case	Country	Programme/technology	Aim of the programme or implementation	Setting and scale
Telehealth and remote monitoring	4	Ireland	Remote monitoring for heart failure patients	To support post-discharge monitoring of patients with heart failure through home-based measurements, patient onboarding, and nurse-led follow-up.	Hospital-to-home care pathway; regional implementation
	5		SMILE 2/ProACT remote monitoring programme for chronic disease management	To support daily self-monitoring, symptom reporting, and clinical escalation for patients with chronic conditions in community-based care.	Community-based chronic care; regional implementation
	6	Denmark	TeleCare North chronic obstructive pulmonary disease (COPD) home monitoring	To support home-based monitoring of patients with chronic obstructive pulmonary disease and strengthen coordination between municipal and hospital services.	Home-based monitoring integrated with municipal and hospital services; regional/large-scale trial
	7		National telehealth and video consultation services	To enable remote consultations and digital communication across primary, secondary, and municipal care services.	Primary, secondary, and municipal care; national implementation
	8		TeleCare North heart failure telemonitoring (Telekit)	To support remote self-monitoring and follow-up for patients with heart failure as part of usual care.	Hospital and home-based care; regional/randomized controlled trial-based implementation
Artificial intelligence-enabled diagnostic, screening, and decision-support systems	10	Germany	Smart glasses and artificial intelligence-supported wound monitoring	To enable remote wound assessment through smart glasses, artificial intelligence-based measurement, and real-time specialist consultation.	Home-based/remote wound care; local implementation
	11	Czech Republic	Artificial intelligence-supported mammography screening (Transpara Breast Care)	To support radiologists in mammography screening through artificial intelligence-based triage and decision support.	Mammography centre/clinic; local implementation
	13		Artificial intelligence retinal screening system (Aireen)	To support retinal image analysis and preventive screening in ophthalmology practice.	Private ophthalmology clinic; local clinical implementation
Extended reality-based therapeutic and rehabilitation interventions	1	Poland	Virtual reality (VR) system for pain and anxiety reduction (Healthy Mind VR)	To provide non-pharmacological support for reducing pain and anxiety during hospital procedures.	Hospital neurology ward; local implementation
	9	Romania	Virtual reality-supported physiotherapy training and rehabilitation	To integrate virtual reality into physiotherapy training and rehabilitation activities, linking student training with patient-facing rehabilitation practice.	Physiotherapy education and rehabilitation practice; local/institutional implementation
Digital therapeutic, self-management, and patient-support platforms	3	Ireland	SilverCloud internet-delivered cognitive behavioural therapy platform	To provide guided internet-delivered cognitive behavioural therapy within public mental health services.	National mental health services; national implementation
Assistive and socially interactive technologies	2	Poland	PARO therapeutic seals	To support therapeutic, psychosocial, pedagogical, and speech-therapy activities for refugee children.	Community-based psychosocial and therapeutic support centres; multi-site local/community implementation
	12	Czech Republic	Protectu remote assistance system for older adults	To provide older adults with emergency support, remote assistance, and call-centre-based support for independent living.	Home-based assistance and social care; commercial deployment

To make the selection process more transparent, all excluded case reports were described in Supplementary Table S1. The table includes the country, technology or intervention type, intended setting, reported implementation status, and main reason for exclusion. Exclusion was based on insufficient evidence of real-world implementation, not on whether the intervention was reported as successful or unsuccessful. It is also important to clarify the nature of the data. The analyzed material did not consist of primary qualitative data collected through interviews, focus groups, observations, or ethnographic fieldwork. The study was based on secondary analysis of standardized implementation case reports prepared by project partners. For this reason, the findings should be interpreted as a structured cross-case synthesis of reported implementation experiences, rather than as an in-depth qualitative reconstruction of implementation processes based on primary data.

### 2.3 Analytical framework

The 13 case studies were analyzed using inductive descriptive coding and cross-case synthesis, in line with multiple case study research (19, 20). The aim was to identify determinants of digital technology implementation that were reported across cases and to organize them into an analytical framework.

The analysis was informed by established implementation frameworks, but these frameworks were not used as coding templates. In particular, the Non-adoption, Abandonment, Scale-up, Spread, and Sustainability (NASSS) framework (5) and the Consolidated Framework for Implementation Research (CFIR) (6) were used as interpretive reference points. The NASSS framework was especially useful because it draws attention to the complexity of digital health implementation, including the technology, adopters, organizations, wider institutional context, and the process of embedding and adapting a solution over time (5). In this study, NASSS and CFIR were not applied as deductive coding schemes. The analysis started with the case reports themselves. Each report was reviewed independently, with attention to statements describing barriers, facilitators, implementation conditions, user experiences, organizational factors, technological issues, system-level influences, and lessons learned. Relevant implementation-related statements were first coded descriptively and then grouped into broader categories. This approach was consistent with thematic analysis, where patterns of meaning are identified and organized into analytical categories (23).

The coding process included five main steps: (i) identifying implementation-related statements in the structured case reports; (ii) coding reported barriers, facilitators, contextual conditions, and lessons learned; (iii) grouping similar codes into thematic categories; (iv) aggregating these categories into higher-order implementation determinants; and (v) organizing the determinants into framework domains.

Clear coding rules were used for the determinant presence matrix. A determinant was coded as “present” when a case report contained clear, case-specific evidence that the determinant was relevant to the implementation process, for example as a barrier, facilitator, condition, resource, requirement, or lesson learned. A determinant was coded as “partially present” when the evidence was indirect, limited, or incomplete. This included cases where the issue was only briefly mentioned, described as a general contextual factor, or implied through implementation challenges but not discussed in detail. If the determinant was not mentioned, or if the information was too limited to support coding, it was coded as “not reported/insufficient evidence”. Two researchers coded determinant presence independently and then discussed differences until consensus was reached.

After coding the individual case reports, determinants were compared across all cases. The purpose was not to measure how strongly each determinant influenced implementation, but to identify patterns in what was reported across different countries, technologies, and levels of healthcare delivery. Determinants with similar functions were grouped together and then organized into broader conceptual categories. This process led to the identification of 20 recurring reported implementation determinants. These were grouped into four domains: technological, organizational, user-related, and system-level. Each domain included five determinants. The four-domain structure was developed from the case material and should not be understood as a direct mapping of NASSS, CFIR, or any other single framework. The domains and determinants are summarized in Table 2.

**Table 2:** Four-domain structure of recurring implementation determinants identified through cross-case analysis of digital health implementations.

Domain	Implementation Determinants
Technology	Interoperability with existing systems; Technological stability and reliability; Data security and privacy; Usability and interface ergonomics; Perceived clinical value
Organization	Leadership, administrative governance, and implementation coordination; Alignment with existing workflows; Organizational infrastructural readiness; Availability of technical support; Interdepartmental and team coordination
User	Digital competencies of healthcare staff and other users; Structured user training and onboarding; Acceptance of technology by professional and patient/end users; Engagement of clinicians, frontline personnel, and end users; Operational workload implications for staff and care teams
System	Funding sustainability; Regulatory and legal compliance; Institutional and policy-level support; Cross-sectoral integration of care; Health system readiness, equity, and digital inclusion

The approach shared some features with framework-informed qualitative analysis, especially at the interpretation stage. However, it was not a formal framework analysis based on an *a priori* coding matrix. The work proceeded in two stages. First, implementation-related statements were coded inductively from the case reports, without assigning them to predefined NASSS or CFIR categories. Second, after the empirical determinant structure had been developed, NASSS and CFIR were used ex post to help interpret the scope of the findings and position the four-domain structure within implementation science literature. In this sense, the study combined inductive descriptive coding with later theoretical interpretation, rather than applying a deductive or mixed deductive–inductive framework analysis from the start.

The resulting framework should therefore not be presented as a new theory competing with existing implementation frameworks. It is also not a formal empirical validation of NASSS or CFIR. Rather, it is a pragmatic synthesis and operationalisation tool that organizes recurring reported determinants into a compact structure that may be useful for implementation planning and readiness assessment (24). To make the ex post use of NASSS and CFIR more transparent, Supplementary Table S3 shows how the four domains and selected determinants were aligned with corresponding domains and constructs from these frameworks. This alignment was used only for interpretation and theoretical positioning, not to generate the original coding categories. To support transparency, selected examples from the case reports are included in the Results section at the domain level. A more detailed set of operational definitions and illustrative evidence for all determinants is provided in Supplementary Table S2.

### 2.4 Rigor and trustworthiness

Several steps were used to strengthen the rigor and trustworthiness of the analysis. First, the case reports were coded independently by two researchers with experience in qualitative analysis and implementation research in healthcare. The analytical team included an academic researcher specializing in public health and a public health practitioner with experience in healthcare innovation. After independent coding, the researchers compared their interpretations. Differences were discussed until agreement was reached. Because the analysis was interpretive in nature, formal inter-coder reliability statistics were not calculated. Second, the analysis drew on case materials from six independent partner institutions working in different healthcare systems and organizational settings. This allowed comparison of implementation experiences across different countries, institutions, and clinical contexts. Third, all case reports were prepared using the same standardized reporting template. This supported comparability between cases. The template included information on implementation context, implementation process, stakeholders, organizational and clinical outcomes, barriers, facilitators, and lessons learned. Fourth, the study used a maximum variation approach. The included cases differed by type of digital technology, level of healthcare delivery, organizational context, implementation scale, and national health system. This variation was important for assessing whether similar implementation issues appeared across different settings.

Transferability was addressed by providing contextual information about the included cases and by showing how determinants were reported across heterogeneous implementations. The study did not aim for statistical generalizability. Instead, it aimed for analytical transferability. That is, to help readers judge whether the findings may be relevant to other digital health implementation contexts. Table 1 describes the included cases, Table 2 presents the determinant structure, and Table 3 shows the presence of reported determinants across cases.

**Table 3:** Presence matrix of reported implementation determinants across the 13 analyzed digital health case reports.

Determinant	C1	C2	C3	C4	C5	C6	C7	C8	C9	C10	C11	C12	C13
Technology domain													
Interoperability with existing systems	—	—	◐	◐	●	●	●	●	—	◐	●	●	◐
Technological stability and reliability	◐	◐	◐	●	●	●	●	◐	●	●	●	●	●
Data security and privacy	—	—	●	●	◐	—	◐	—	—	—	◐	—	—
Usability and interface ergonomics	●	●	●	●	●	◐	●	◐	●	◐	●	●	●
Perceived clinical value	●	●	●	●	●	◐	●	◐	●	●	●	●	●
Organizational domain													
Leadership, administrative governance, and implementation coordination	◐	●	●	●	●	●	●	●	◐	◐	●	◐	●
Alignment with existing workflows	●	●	●	●	●	●	●	●	◐	●	●	●	●
Organizational infrastructural readiness	◐	◐	◐	●	●	●	●	●	●	●	●	●	●
Availability of technical support	◐	◐	●	●	●	●	●	●	●	●	●	●	●
Interdepartmental and team coordination	◐	●	●	●	●	●	●	●	●	●	●	●	●
User-related domain													
Digital competencies of healthcare staff and other users	●	◐	●	●	●	●	●	●	●	●	●	◐	●
Structured user training and onboarding	●	●	●	●	●	●	●	●	●	●	●	—	●
Acceptance of technology by professional and patient/end users	●	●	●	●	●	◐	◐	◐	●	●	●	◐	●
Engagement of clinicians, frontline personnel, and end users	◐	●	●	●	●	●	●	●	◐	●	●	◐	●
Operational workload implications for staff and care teams	●	◐	●	◐	●	◐	◐	◐	—	●	●	●	●
System-level domain													
Funding sustainability	◐	◐	●	●	●	●	●	●	—	—	●	●	●
Regulatory and legal compliance	◐	—	●	●	●	—	◐	—	—	—	◐	—	—
Institutional and policy-level support	◐	●	●	●	●	●	●	●	◐	◐	●	◐	◐
Cross-sectoral integration of care	—	●	●	◐	●	●	●	●	—	◐	◐	●	◐
Health system readiness, equity, and digital inclusion	◐	◐	●	◐	●	●	●	●	◐	◐	●	◐	◐

● present = clear case-specific evidence that the determinant was relevant to implementation; ◐ partially present = indirect, limited, or incomplete evidence; — not reported/insufficient evidence = no explicit or sufficient information in the case report to support coding.

### 2.5 Ethical considerations

The study was based on secondary analysis of standardized case study materials developed within the Erasmus+ project “Hybrid Healthcare—Advancing Hybrid Management and Digital Technology in Healthcare Education”. The analysis did not involve direct contact with patients, healthcare professionals, or other participants. No new empirical data were collected, and no identifiable personal data were processed. For this reason, formal ethical approval was not required under institutional and national regulations. Although formal approval was not required, ethical issues were considered during the analysis. The case materials were analyzed in an aggregated and non-identifiable form. No individual patients, healthcare professionals, or institutions were identifiable in the results. The analysis focused on implementation processes, organizational conditions, barriers, facilitators, and lessons learned, rather than on individual clinical or personal data. The materials were used only for the scientific aims of the study and were handled with attention to confidentiality, responsible data use, and research integrity.

## 3 Results

The analysis included thirteen case reports on digital technology implementation in healthcare. The cases differed in terms of technology type, country, healthcare setting, and scale of implementation. Despite this variation, several similar implementation issues appeared across the reports. These issues were organized into four domains: technological, organizational, user-related, and system-level. The domains were developed from the case reports and were later interpreted in relation to the NASSS (5) and CFIR (6) frameworks. The included cases, including their countries, technology classes, programme aims, settings, and scale, are summarized in Table 1.

### 3.1 Recurring determinants of implementation in the analyzed cases

The analysis of the 13 case reports identified 20 recurring implementation determinants. These determinants were reported across different clinical, organizational, and national contexts. They were grouped into four domains: technological, organizational, user-related, and system-level. The four-domain structure and the determinants assigned to each domain are presented in Table 2. Selected examples from the case reports are used below to illustrate the domain-level findings. More detailed definitions and coding evidence for each determinant are provided in Supplementary Table S2.

#### 3.1.1 Technological domain

The technological domain included determinants related to how the digital solution functioned and how easily it could be used in practice. These determinants were interoperability with existing systems, technological stability and reliability, data security and privacy, usability and interface ergonomics, and perceived clinical value (Table 2). In several cases, implementation was affected not by the idea of the technology itself, but by practical issues of use and integration. For example, in the heart failure remote monitoring case, some older patients had difficulties using smartphones, which showed the importance of interface accessibility and fit between the technology and its users (See Table 1).

#### 3.1.2 Organizational domain

The organizational domain included determinants related to how implementation was managed within institutions and care pathways. These determinants were leadership, administrative governance and implementation coordination, alignment with existing workflows, organizational infrastructural readiness, availability of technical support, and interdepartmental and team coordination (Table 2). Organizational issues were especially visible in telehealth and remote monitoring cases. These implementations required not only technical infrastructure, but also clear procedures for patient identification, onboarding, monitoring, follow-up, documentation, and allocation of staff time. For example, the heart failure remote monitoring case showed the importance of embedding remote monitoring into post-discharge care pathways, rather than treating it as an additional activity outside routine care (See Table 1). Administrative issues were therefore treated as part of the organizational domain. They were closely linked to workflow alignment, implementation governance, reimbursement arrangements, and coordination between clinical and organizational actors.

#### 3.1.3 User-related domain

The user-related domain included determinants connected with both professional users and patient or end users, where these perspectives were reported in the case materials. Professional users included healthcare professionals, frontline staff, and implementation teams. Patient and end users included patients, caregivers, older adults, and service users. The main determinants in this domain were digital competencies of healthcare staff and other users, structured user training and onboarding, acceptance of technology by professional and patient/end users, engagement of clinicians, frontline personnel, and end users, and operational workload implications for staff and care teams (Table 2). Human factors were important in translating technological potential into actual use. In several cases, implementation depended on whether users had enough confidence, support, and practical skills to work with the technology. For example, in the SMILE 2/ProACT remote monitoring programme, digital literacy among older or rural patients was reported as a challenge for adoption and sustained engagement (See Table 1). The case reports provided more detail on professional users than on direct patient narratives. Still, patient and end-user perspectives were reflected in reported issues such as usability, digital literacy, engagement, accessibility, and sustained use. Examples included difficulties among older patients using smartphones in remote monitoring, digital literacy challenges among older or rural patients, and the need to adapt technologies to the abilities and expectations of vulnerable or non-specialist users.

#### 3.1.4 System-level domain

The system-level domain included determinants related to the wider healthcare system and institutional environment. These determinants were funding sustainability, regulatory and legal compliance, institutional and policy-level support, cross-sectoral integration of care, and health system readiness, equity, and digital inclusion (Table 2). System-level issues influenced whether digital solutions could be scaled, sustained, or used consistently across settings. In the heart failure remote monitoring case, limited broadband access in rural areas was reported as a barrier to consistent implementation, showing how digital infrastructure can affect the feasibility of remote care (See Table 1). Equity-related issues were included in this domain because they were linked to access, infrastructure, and readiness for digital care. They appeared in reports describing rural connectivity problems, digital literacy barriers among older or rural patients, and the risk that digital health could widen rather than reduce inequalities. However, equity was not reported with the same depth across all case reports. For this reason, it was treated as a cross-cutting system-level issue rather than as a separate determinant.

### 3.2 Patterns of recurrence across cases

The cross-case synthesis showed that some determinants were reported in many case reports, while others appeared less consistently. The presence matrix should be read as a descriptive summary of reported evidence, not as a measure of how strong or causally important each determinant was (Table 3). The most frequently reported determinants included perceived clinical value, alignment with existing workflows, acceptance of technology by professional and patient/end users, structured user training and onboarding, availability of technical support, and administrative, interdepartmental, and team coordination. These issues appeared across different technologies and settings, suggesting that they were common implementation concerns in the analyzed material. Other determinants were also reported, but their presence varied more by technology type and context. This was the case for interoperability, technological stability, organizational infrastructural readiness, funding sustainability, and institutional or policy-level support. Data security and privacy, regulatory and legal compliance, cross-sectoral integration of care, operational workload implications for staff and care teams, and health system readiness, equity, and digital inclusion were reported less evenly across the cases. For this reason, Table 3 should not be interpreted as a ranking of determinant importance, but as a transparent summary of what was reported in the available case materials.

### 3.3 Development of the four-domain analytical framework

After coding and comparing the case reports, determinants with similar functions were grouped into four domains: technological, organizational, user-related, and system-level. Each domain included five determinants, giving a total of 20 implementation determinants. The four-domain structure was developed from the case material and was then interpreted in relation to existing implementation frameworks. Table 3 shows how the reported determinants appeared across the analyzed cases. All four domains were represented in the case reports, although the individual determinants were not reported with the same frequency or level of detail. This supports the use of the framework as a way to organize implementation issues across different digital health contexts. The ex post alignment with NASSS (5) and CFIR (6) is presented in Supplementary Table S3. This mapping was used to show how the domains developed in this study relate to established implementation concepts. It was not used to create the original coding categories. The results address the aim of the study by identifying recurring reported implementation determinants and organizing them into a four-domain analytical framework. The framework may be useful as an analytical and planning tool, but its transferability should be examined in future studies.

## 4 Discussion

The aim of this study was to identify implementation determinants reported across diverse digital health cases and to organize them into a four-domain analytical framework covering technological, organizational, user-related, and system-level areas. The cross-case synthesis showed that, despite variation in technology type, country, healthcare setting, and implementation scale, several issues appeared repeatedly across the case reports. These findings support the view that digital health implementation is rarely determined by the technical solution alone. More often, implementation problems emerge where a digital tool meets existing clinical routines, organizational capacity, professional roles, user expectations, infrastructure, and policy conditions. The analysis identified 20 determinants across the case reports. Their recurrence does not imply that each determinant had the same weight in every case, nor that the framework can predict implementation success. Rather, the findings show which types of issues were repeatedly visible in the available implementation narratives. In this sense, the framework provides a structured way to read across heterogeneous digital health implementations and to identify areas that may require attention before or during implementation. This is consistent with previous reviews showing that eHealth and digital health implementation is influenced by multiple interacting factors rather than by single barriers or facilitators alone (8, 9).

### 4.1 Structural nature of implementation determinants

The four domains identified in this study should not be interpreted as separate explanations of implementation. Their value lies in showing how different layers of implementation interact. A digital health solution may be clinically promising and technically reliable, but still difficult to embed if it disrupts workflows, increases staff workload, lacks technical support, or is introduced into a system without stable funding or regulatory clarity. Conversely, even a relatively simple technology may be adopted more easily when it fits existing care pathways, is supported by leadership, and responds to a clearly perceived clinical need. This interpretation is consistent with previous implementation research showing that digital health adoption is shaped by many interacting factors (5, 6, 8, 9, 13, 25). However, the present study adds a cross-case perspective by showing how these issues appeared across different types of digital health technologies, including telemonitoring, artificial intelligence-enabled tools, extended reality solutions, digital platforms, and assistive technologies. The recurrence of workflow alignment, user acceptance, training, technical support, and perceived clinical value suggests that implementation teams should treat these areas as early warning points, even when the technology itself differs substantially between projects. The findings also highlight the central role of organizational conditions. In several cases, implementation required not only technical installation, but also changes in procedures, documentation, patient onboarding, follow-up routines, staff responsibilities, and coordination between teams. This supports the view that digital transformation in healthcare is an institutional process rather than a purely technical intervention. Technologies become sustainable only when they are translated into everyday organizational work (1, 2).

### 4.2 Implementation as a socio-organizational process

The cases also show that implementation depends strongly on how professionals, patients, and other end users experience the technology in practice. User-related determinants, such as digital competencies, training and onboarding, acceptance, engagement, and workload implications, were not secondary issues. They influenced whether digital solutions could move from formal availability to actual use. This is consistent with implementation frameworks showing that adoption is shaped by the interaction between the technology, adopters, organizations, and the wider implementation context (5, 6, 8).

This is particularly important in healthcare, where digital tools often alter established clinical routines, professional roles, and relationships between care providers and patients. A solution may be perceived as useful at the strategic level, but resisted or underused if frontline staff do not see its value, if it adds administrative burden, or if users are not sufficiently supported. Previous research on technology acceptance in healthcare similarly shows that perceived usefulness, ease of use, trust, and user competencies influence whether digital solutions are accepted and used over time (26, 27).

Similarly, patients and caregivers may benefit from remote monitoring or digital platforms only when the technology is accessible, understandable, and adapted to their abilities and everyday circumstances. The case reports provided more detail on professional and organizational perspectives than on direct patient narratives. Nevertheless, patient and end-user issues were visible through reported barriers related to usability, digital literacy, accessibility, rural connectivity, and sustained engagement. These findings are in line with studies showing that digital literacy, accessibility, support, and confidence in using technology may influence sustained use, particularly among older adults, rural populations, and users with chronic diseases or limited digital confidence (11, 12).

The findings also suggest that the relative importance of user-related and organizational determinants may vary between health systems. For example, implementations in highly digitalized settings, such as Denmark (See Table 1), could build on stronger digital infrastructure, interoperability standards, national identifiers, and policy support for digital health. In other contexts, the case reports more often pointed to challenges related to digital literacy, infrastructure variability, organizational readiness, and user support. This means that the proposed framework should not be applied mechanically. It can help organize recurring implementation issues, but its use in practice requires attention to the local setting, including health system structure, regulatory maturity, organizational culture, and attitudes toward digital technologies.

The results therefore suggest that implementation planning should not treat “users” as a single homogeneous group. Healthcare professionals, patients, caregivers, technical staff, managers, and commissioners may each experience the same digital solution differently. For implementation teams, this means that user engagement should begin before deployment and continue after the technology enters routine practice. In this sense, implementation is not simply a technical roll-out, but a socio-organizational process in which technology becomes usable only when it is aligned with professional practice, patient needs, organizational routines, and available support.

### 4.3 Coherence of the domain framework and its theoretical significance

The four-domain structure proposed in this study is broadly consistent with previous implementation research, which shows that healthcare implementation depends on several interacting levels rather than on one isolated factor (5–8). In this study, technological, organizational, user-related, and system-level issues repeatedly appeared together. This does not mean that the domains explain implementation in a causal way. Rather, they provide a practical structure for organizing the issues reported across different digital health implementation contexts. At the same time, the framework should be positioned carefully. The proposed framework differs from broader conceptual frameworks such as NASSS (5) and CFIR (6). It was developed from a structured cross-case synthesis of reported real-world implementation experiences, not from theory alone. It should therefore not be read as a fully inductive explanatory model, a new theory of digital health implementation, or a formal validation of existing implementation frameworks (5, 6, 28). Rather, it is an empirically informed structure that organizes recurring reported implementation issues into a form that may be useful for planning and readiness assessment. Its explanatory and predictive value still needs to be tested in future studies. This use of cross-case synthesis is consistent with multiple case study research, which supports comparison across cases and the identification of patterns across contexts (19, 20).

The practical value of the framework lies in its ability to draw attention to interdependencies between determinants. For example, interoperability is not only a technical issue if it affects documentation, communication between services, workload, and continuity of care. Similarly, user training is not only an educational issue if it influences professional confidence and willingness to change established routines. Funding sustainability is not only a financial issue if it determines whether staff support, technical maintenance, and scale-up can continue after the initial implementation phase. In this way, the framework may help implementation teams move beyond a simple list of barriers and consider how different implementation conditions reinforce or weaken one another. This is also consistent with implementation research that distinguishes implementation processes from implementation outcomes and emphasizes the need to define implementation factors clearly before assessing their effects (29). From a broader health system perspective, the framework connects micro-, meso-, and macro-level issues: users and clinical practice, organizational routines and governance, technology characteristics, and wider system conditions. This aligns with digital health policy and health system literature, which emphasizes that digital transformation requires alignment between technology, organizations, users, infrastructure, and system-level conditions (1, 2).

### 4.4 Practical implications for implementation planning

From a practical perspective, the proposed framework may help teams plan digital health implementation in a more structured way. It can be used to assess readiness before implementation and to identify areas that may require additional preparation. These areas may include technical infrastructure, workflow integration, staff competencies, user support, governance, funding, and regulatory conditions. Such preparatory work is important because implementation problems often become visible only after a technology is introduced into routine practice. Reporting guidelines and implementation planning tools can support this process by making implementation assumptions, risks, and decisions more explicit (21).

The four-domain structure may also help implementation teams avoid focusing too narrowly on the technology itself. A digital solution may be technically sound, but still difficult to implement if it does not fit existing workflows, if staff are not prepared to use it, or if technical support is insufficient. For this reason, the framework can be used as a diagnostic tool by healthcare institutions, implementation teams, and policymakers to map potential barriers and resources across technological, organizational, user-related, and system-level domains (25).

The findings also suggest that implementation should be planned as an integrated process. Purchasing or introducing a digital tool is only one part of implementation. Other elements, such as adapting procedures, preparing staff, organizing communication, securing funding, and clarifying regulatory requirements, are also needed. This is consistent with previous literature on eHealth implementation, which shows that organizational transformation and alignment with routine care are often necessary for sustainable digital health adoption (5, 9).

The framework may therefore support the design of implementation strategies by helping teams decide where action is needed first. It can be used to structure readiness checklists, map organizational barriers, and plan training and communication activities. Such strategies should be adapted to the specific organizational context and developed with the involvement of relevant stakeholders, including healthcare professionals, managers, technical teams, and, where appropriate, patients or end users (13). At the system level, the framework may help identify risks that affect sustainability and scale-up. These include unstable funding, weak infrastructure, limited interoperability, unclear regulations, and unequal access to digital services. Considering these issues early may support more sustainable digital transformation and may also help reduce the risk that digital health innovations increase existing inequalities (11, 12). Strengthening implementation capacity at organizational and system levels may also contribute to more resilient healthcare systems, especially in the context of growing service demand, workforce shortages, and financial pressure (1, 2).

### 4.5 Limitations and strengths of the study

This study has several limitations. The case reports came from one international Erasmus+ project. Although the cases differed by country, technology type, healthcare setting, and implementation scale, the sample was still purposive. It may therefore include more mature or better documented implementations, because these were easier for project partners to report. To make this clearer, the excluded cases are described in Supplementary Table S1. However, some selection bias toward more developed or better documented cases cannot be fully ruled out. Next, the study was based on secondary standardized case reports, not on primary qualitative data. The case descriptions differed in depth and detail. This limited the possibility of checking reported implementation experiences directly and may have introduced selective reporting of barriers, facilitators, contextual conditions, and lessons learned. It also affected the representation of patients and end users. Some reports included information on usability, digital literacy, accessibility, engagement, or sustained use, but the material more often reflected the perspective of healthcare professionals, implementation teams, and organizational actors than direct patient narratives. Moreover, the cases were heterogeneous. They differed in technology type, healthcare setting, organization, and national system. This heterogeneity was useful for identifying recurring reported issues, but it also limited direct comparison between cases. The findings should therefore be interpreted as a descriptive synthesis of reported determinants, not as a quantitative assessment of their relative strength or causal influence. Finally, the proposed framework has its own limitations. It summarizes determinants that were repeatedly reported across different implementation contexts, but it does not assign weights to individual determinants, model causal relationships, or predict implementation outcomes. It should therefore be used as an analytical and planning tool, not as a prescriptive model. Its use requires adaptation to the specific technological, organizational, cultural, and system-level context. Future studies should validate the framework in broader healthcare settings and use mixed-methods designs to examine the relative influence of individual determinants and to include patient, caregiver, and end-user perspectives more directly.

The study also has several strengths. It brings together real-world implementation experiences from six European countries and different healthcare systems. The cases covered several types of digital health technologies and levels of care. The use of a standardized case reporting template improved comparability between cases, while the cross-case synthesis made it possible to identify implementation issues that appeared across different contexts. The study also provides a practical framework that may help organize implementation knowledge and support readiness assessment when digital health technologies are prepared for routine practice.

### 4.6 Directions for future research

Future research should test the framework in a broader and more diverse set of digital health implementations. In particular, it would be useful to examine whether the framework can help assess implementation readiness and anticipate potential barriers before implementation begins. Further studies should also use mixed-methods designs. Qualitative research could explore how the determinants operate in specific organizational settings, while quantitative studies could examine their relative importance and relationships between domains. This would also support the development of practical measurement tools, such as readiness scales, implementation risk assessment instruments, or structured checklists based on the four-domain framework. Another important direction is to examine how system-level and cultural conditions shape digital health implementation. Cross-country studies and comparisons across different levels of healthcare systems could help assess whether the framework is transferable and how it needs to be adapted to different institutional, regulatory, and organizational contexts.

## 5 Conclusions

This study identified recurring determinants of digital technology implementation in healthcare and organized them into four domains: technological, organizational, user-related, and system-level. The findings suggest that implementation is not shaped by technology alone. It also depends on how well the solution fits organizational routines, user needs, available resources, and wider system conditions. The proposed four-domain framework offers a practical way to organize reported implementation issues across different digital health contexts. It may help implementation teams assess readiness, identify risk areas, and plan actions before or during implementation. In this sense, the framework can support more structured preparation for digital transformation in healthcare. The framework should not be treated as a predictive model of implementation success. Rather, it is an analytical and planning tool based on reported real-world implementation experiences. Further studies are needed to test its usefulness in other healthcare systems and to examine how individual determinants influence implementation outcomes.

## Data Availability

The data analyzed in this study is subject to the following licenses/restrictions: The datasets analyzed in this study originate from institutional case reports developed within an Erasmus+ project and partner organizations. Due to institutional agreements, data ownership, and confidentiality considerations, the datasets are not publicly available. Access to the data may be granted upon reasonable request and subject to approval by the respective institutions. Requests to access these datasets should be directed to mariusz.jaworski@wum.edu.pl.
