# Supplementary material for: Recurring implementation determinants in digital health innovations: a multi-context multiple case study and cross-case synthesis into a four-domain analytical framework
Source: Front Digit Health. 2026 Jul 15;8:1844330. doi: 10.3389/fdgth.2026.1844330 (PMC13416340; doi:10.3389/fdgth.2026.1844330)
Supplement: Supplementary file 1 [file Datasheet1.pdf]

Supplementary Table S1. Characteristics of excluded case reports and reasons for exclusion

| ID | Case report title / technology | Country | Intended setting | Reported implementation status | Primary reason for exclusion |
|---|---|---|---|---|---|
| E1 | MedQuiz Professional Designer | Poland | University and clinical education | Custom Generative Pre-trained Transformer (GPT) tool used to support quiz and assessment design | Excluded because the case focused on medical education and assessment development rather than implementation of a digital health solution in healthcare delivery or routine clinical practice. |
| E2 | Virtual Patient: Enhancing Clinical Reasoning Skills in Midwifery Education | Poland | University and clinical education | Educational application used by students to support clinical reasoning and care planning | Excluded because the case was primarily education-focused, with students as the main users, and did not represent implementation of a digital health intervention as part of routine healthcare service delivery. |
| E3 | Interactive Platform for Clinical Trials | Romania | Research and education institution | Platform concept/design to connect volunteers with clinical trial sponsors | Excluded because the case described a proposed or planning-stage digital platform, with insufficient evidence of routine real-world implementation in healthcare practice. |
| E4 | Enhancing Medical Training through Clinical Simulation | Romania | Medical university simulation centre | Simulation system integrated into medical training and assessment | Excluded because the case described simulation-based education rather than implementation of a digital health |

| | | | | | |
|---|---|---|---|---|---|
| | | | | | intervention in real-world clinical or organizational practice. |
| E5 | Enhancing Medical Teaching by Using Smart Boards | Romania | Medical teaching infrastructure | Smart boards introduced into teaching settings | Excluded because the case focused on educational infrastructure and teaching enhancement, not on implementation of digital health technology in healthcare delivery. |
| E6 | DiaMáma digital education and self-management app for gestational diabetes | Czech Republic | Home-based / outpatient care support | Pilot evaluation and test operation with end users | Excluded because the case was primarily a digital education and self-management support tool at pilot/test-operation stage, with limited evidence of sustained integration into routine outpatient care pathways. |
| E7 | Wexford Telehealth Pilot Project | Ireland | Community-based healthcare pilot | Twelve-week telehealth pilot for patients with chronic conditions | Excluded because the case described a short, time-limited pilot with limited evidence of sustained integration into routine care pathways or long-term implementation. |
| E8 | Artificial intelligence (AI) Decision-Support in Emergency Department, Odense University Hospital | Denmark | Hospital emergency department | Ongoing artificial intelligence (AI) decision-support project with initial pilot sub-projects | Excluded because the case was still ongoing and provided insufficient evidence of routine, sustained implementation in everyday emergency department practice. |
| E9 | Artificial intelligence (AI)-powered drones | Germany | Research university / | Experimental drone and wearable health | Excluded because the case described prototype or experimental testing rather |

| | | | | | |
|---|---|---|---|---|---|
| | and wearable health devices for emergency medical response | | campus-based testing | monitoring system tested under controlled campus conditions | than routine deployment in emergency medical services or healthcare delivery. |
| E10 | VISAKI – Virtually Alleviating Social Anxiety Disorders in Children | Germany | Higher education and business consortium / planned therapeutic context | Virtual reality (VR) and gamification platform under development, with functional prototype and planned pilot testing | Excluded because the case was at the development/prototype stage and had not yet been implemented as a routine therapeutic intervention in clinical practice. |
| E11 | Health Data Analytics Course | Germany | Higher education / online course | AI-based tutor integrated into an academic course, with expected pilot evaluation among students | Excluded because the case was education-focused and concerned AI-supported learning rather than implementation of a digital health solution in healthcare practice. |

Note. ID - Excluded case ID

Supplementary Table S2. Operational definitions and illustrative evidence for implementation determinants

| Domain | Determinant | Operational coding definition | Evidence required for coding as "present" | Illustrative evidence from included case reports |
|---|---|---|---|---|
| Technology | Interoperability with existing systems | The extent to which the digital solution required, enabled, or depended on integration with existing clinical, administrative, communication, monitoring, or data systems. | Clear evidence that the technology exchanged data with, was embedded in, or was dependent on existing clinical, organizational, municipal, national, or digital health infrastructure. | C4: home monitoring data were transmitted from Bluetooth-enabled devices and a patient app to specialist nurse teams. C5: the ProACT platform supported daily patient self-checks and real-time clinical monitoring. C7: national telehealth relied on digital messaging systems, video consultation platforms, and national eHealth infrastructure. |
| | Technological stability and reliability | The degree to which the digital solution functioned consistently, safely, and dependably during implementation, including hardware, software, connectivity, and | Clear evidence that technical reliability, connectivity, equipment maintenance, software stability, device performance, or system continuity affected implementation. | C1: VR use required device sanitation and maintenance in the hospital ward. C10: wound monitoring depended on reliable smart glasses, AI measurement software, and 5G connectivity. C12: Protectu required uninterrupted 24/7 |

| | | | | |
|---|---|---|---|---|
| | | maintenance requirements. | | operation and reliable SOS/wearable devices connected to a call centre. |
| | Data security and privacy | The relevance of data protection, privacy, cybersecurity, consent, secure data storage, General Data Protection Regulation (GDPR) compliance, or confidential handling of health information during implementation. | Clear evidence that privacy, General Data Protection Regulation (GDPR), cybersecurity, consent, secure cloud storage, or ethical data handling was reported as an implementation requirement, barrier, or condition. | C4: data security was handled through GDPR-compliant cloud storage and informed consent procedures. C3: digital mental health implementation required data privacy and clinician confidence in interpreting digital engagement data. C13: AI retinal screening involved automated analysis of patient retinal images in a clinical setting, requiring secure handling of health data. |
| | Usability and interface ergonomics | The extent to which the digital solution was perceived as intuitive, accessible, easy to use, and suitable for the target users, including patients, professionals, caregivers, | Clear evidence that ease of use, intuitive interface, accessibility, user-friendliness, or usability-related difficulties affected uptake or routine use. | C1: Healthy Mind VR was described as having an intuitive interface and quick preparation process. C4: some older patients struggled with smartphone usage in remote monitoring. |

| | | | | |
|---|---|---|---|---|
| | | and implementation teams. | | C5: user-friendly digital tools and onboarding supported patient engagement. C13: Aireen could evaluate retinal images rapidly and could be used by less specialized healthcare staff. |
| | Perceived clinical value | The extent to which the technology was reported as clinically useful, beneficial for care quality, supportive of diagnosis or therapy, or meaningful for patient outcomes and professional decision-making. | Clear evidence that the digital solution was perceived to improve care, support clinical decisions, reduce symptoms, increase access, improve monitoring, or strengthen patient management. | C1: VR reduced perceived pain and anxiety during procedures. C3: SilverCloud improved access to guided internet-delivered cognitive behavioural therapy (iCBT) and showed clinically significant improvement among users. C11: AI mammography increased radiologists' diagnostic confidence and supported triage of higher-risk cases. C13: Aireen increased preventive screening capacity in ophthalmology. |
| Organization | Leadership, administrative | The presence of organizational leadership, | Clear evidence that leadership, management, | C2: implementation relied on collaboration between |

| | | | | |
|---|---|---|---|---|
| | governance, and implementation coordination | managerial decision-making, coordination structures, implementation oversight, or governance arrangements supporting adoption and use. | steering groups, project governance, institutional decision-making, or implementation leadership shaped the implementation process. | the Medical University of Warsaw, UNICEF Spilno Hubs, WCPR, and therapeutic staff. C7: national telehealth implementation involved national authorities, regions, municipalities, general practitioners (GPs) and governance arrangements. C11: AI mammography implementation was supported by management of the mammography centre and a clear implementation decision. |
| | Alignment with existing workflows | The degree to which the digital solution was embedded in or adapted to existing clinical, therapeutic, care, organizational, or service delivery processes. | Clear evidence that the technology was integrated into routine care pathways, clinical work, therapy sessions, home-care procedures, or service workflows. | C1: Healthy Mind VR was integrated into routine ward practice during procedures associated with pain or anxiety. C4: remote monitoring was embedded into post-discharge heart failure care pathways. C5: SMILE 2 used daily self-monitoring and escalation |

| | | | | |
|---|---|---|---|---|
| | | | | protocols within community chronic care. C11: AI mammography was integrated into radiology workflow. |
| | Organizational infrastructural readiness | The availability of infrastructure, equipment, IT systems, connectivity, staff capacity, service structures, and organizational resources needed to implement the solution. | Clear evidence that infrastructure, equipment, connectivity, organizational resources, IT capacity, devices, or service structures supported or constrained implementation. | C6: TeleCare North required home-monitoring kits, data infrastructure, staff training, and municipal–hospital coordination. C7: national telehealth benefited from Denmark's digital infrastructure and national identifiers. C10: wound monitoring depended on smart glasses, AI software, 5G internet, and remote specialist access. C12: Protectu required a 24/7 call centre and reliable devices. |
| | Availability of technical support | The presence of technical assistance, troubleshooting, maintenance, information technology (IT) support, | Clear evidence that implementation required or benefited from technical assistance, device support, software support, | C4: older patients were supported through in-person onboarding and telephone support. C5: scalability required ongoing |

| | | | | |
|---|---|---|---|---|
| | | onboarding support, vendor support, or professional assistance enabling sustained technology use. | troubleshooting, training support, or ongoing maintenance. | training and technical support. C10: reliable wound monitoring required training of wound managers and technical optimization of AI software. C12: Protectu required continuous operational and staffing support. |
| | Interdepartmental and team coordination | The extent to which implementation depended on coordination between clinical teams, departments, municipalities, technology providers, academic partners, social care actors, or other stakeholders. | Clear evidence of multidisciplinary, interdepartmental, cross-organizational, or cross-sector coordination in planning, implementation, monitoring, or service delivery. | C2: PARO implementation involved psychologists, pedagogists, speech therapists, UNICEF hubs, WCPR, and university coordination. C4: St. Vincent's remote monitoring involved cardiology teams, specialist nurses, patientMpower, and Health Service Executive (HSE) digital transformation actors. C7: national telehealth required coordination between national authorities, regions, municipalities, general practitioners (GPs) |

| | | | | |
|---|---|---|---|---|
| | | | | home-care services, patients, and IT vendors. |
| User | Digital competencies of healthcare staff and other users | The digital skills, confidence, readiness, and ability of healthcare staff, patients, caregivers, and other users to operate, interpret, or engage with the technology. | Clear evidence that staff digital literacy, confidence, training needs, resistance, or ability to use the technology influenced implementation. | C1: staff scepticism and digital literacy were identified as implementation challenges. C4: investment in digital literacy training for patients and staff was recommended. C9: educators required support to adopt VR tools in physiotherapy. C10: healthcare professionals required training to use smart glasses and AI wound measurement. |
| | Structured user training and onboarding | Formal or informal preparation, onboarding, instruction, or training for professionals, patients, caregivers, students, or implementation teams before or during implementation. | Clear evidence of training sessions, onboarding, preparation materials, staff instruction, patient education, or practical demonstrations supporting use of the technology. | C1: staff were trained to use Healthy Mind VR, adjust session content, and maintain device hygiene. C4: patients received onboarding from specialist nurses. C5: SMILE 2 included patient training sessions. C11: AI |

| | | | | |
|---|---|---|---|---|
| | | | | mammography implementation included staff training after system configuration. |
| | Acceptance of technology by professional and patient/end users | The willingness, trust, satisfaction, engagement, or positive perception of patients, professionals, caregivers, or other users toward the technology. | Clear evidence that users accepted, trusted, valued, were satisfied with, or engaged with the technology, or that resistance/scepticism affected implementation. | C1: patients and nurses reported positive experiences with VR during hospital procedures. C2: professionals rated PARO highly in group and individual therapeutic processes. C3: SilverCloud achieved high user satisfaction. C11: radiologists initially showed scepticism but acceptance improved through gradual staff-centred introduction. |
| | Engagement of clinicians, frontline personnel, and end users | The active involvement of healthcare professionals, frontline staff, patients, caregivers, or other end users in implementing, adapting, using, or sustaining the technology. | Clear evidence that clinicians or frontline staff were actively involved as users, implementers, trainers, co-designers, or evaluators. | C1: nurses used VR in ward routines and provided implementation feedback. C2: psychologists, pedagogists, and speech therapists integrated PARO into therapeutic work. C4: specialist nurses monitored |

| | | | | |
|---|---|---|---|---|
| | | | | heart failure patients remotely. C11: radiologists and technologists participated in AI mammography implementation. |
| | Operational workload implications for staff and care teams | The impact of the technology on staff workload, task distribution, time efficiency, burden, responsibilities, monitoring demands, or workflow efficiency. | Clear evidence that the technology reduced, increased, redistributed, or otherwise changed the workload of professionals or care teams. | C1: VR was reported as time-efficient and facilitated procedures by calming patients. C3: internet-delivered cognitive behavioural therapy (iCBT) helped expand access and reduce pressure on clinicians. C4: remote monitoring required specialist nurse review but aimed to reduce unplanned admissions. C11: AI mammography improved workflow by helping radiologists focus on high-risk cases. |
| System | Funding sustainability | The availability, source, continuity, or sustainability of financial | Clear evidence that funding, reimbursement, grants, public budgets, co- | C1: Healthy Mind VR equipment was funded through a partnership with a |

| | | | | |
|---|---|---|---|---|
| | | resources needed for implementation, operation, scaling, maintenance, or reimbursement. | payments, financial models, or long-term cost sustainability were relevant to implementation. | pharmaceutical company. C3: SilverCloud was publicly funded through the Health Service Executive (HSE). C6 and C8: TeleCare North implementations involved regional or academic research funding and raised cost-effectiveness considerations. C11: AI mammography required a sustainable financial model, resolved through patient co-payments. |
| | Regulatory and legal compliance | The relevance of legal, ethical, medical device, General Data Protection Regulation (GDPR), data governance, certification, reimbursement, liability, or compliance requirements for implementation. | Clear evidence that implementation required compliance with legal, regulatory, ethical, certification, or data governance frameworks. | C1: Conformité Européenne and International Organization for Standardization (ISO) certifications supported institutional acceptance of Healthy Mind VR. C4: informed consent and data privacy protocols were ensured. C5: regulatory and data governance frameworks supported |

| | | | | |
|---|---|---|---|---|
| | | | | implementation. C11: AI use in mammography required ethical and transparent communication with patients. |
| | Institutional and policy-level support | Support from public authorities, healthcare organizations, policy strategies, national or regional programmes, institutional partnerships, or system-level priorities. | Clear evidence that implementation was enabled or shaped by institutional endorsement, public strategy, national/regional health policy, organizational priorities, or partnerships. | C3: SilverCloud aligned with Ireland's national mental health strategy. C5: SMILE 2 was implemented under the Health Service Executive (HSE) Enhanced Community Care programme. C7: national telehealth was supported by Danish policy, regions, municipalities, and public investment. C13: Aireen was implemented in a private clinic as part of a preventive care package. |
| | Cross-sectoral integration of care | The extent to which the solution connected services, sectors, or actors across healthcare, social care, community care, home care, | Clear evidence of integration across sectors, care levels, institutions, or stakeholder groups beyond a single clinical unit. | C2: PARO connected university actors, UNICEF hubs, municipal refugee centres, and therapeutic professionals. C5: SMILE 2 involved Health Service |

| | | | | |
|---|---|---|---|---|
| | | municipal care, technology providers, or family/caregiver networks. | | Executive (HSE), Caredoc, specialist nurses, general practitioners (GPs) and community chronic disease teams. C7: national telehealth linked primary, secondary, municipal, and home-care services. C12: Protectu connected older adults, families, call-centre operators, mobile operators, and social assistance services. |
| | Health system readiness, equity, and digital inclusion | The broader readiness of the health or care system to adopt, scale, sustain, or adapt digital innovations, including digital maturity, infrastructure, workforce capacity, policy environment, cultural readiness, equity considerations, access to digital care, and digital inclusion. | Clear evidence that national or local digital maturity, infrastructure variability, workforce capacity, system pressures, market readiness, or adoption culture influenced implementation. | C6 and C7: Denmark's high digital maturity and infrastructure supported telehealth implementation, although uptake varied across municipalities. C4: rural broadband limitations affected implementation consistency. C10: rural connectivity constraints limited the feasibility of smart-glasses wound monitoring. C12: Protectu faced low market readiness |

| | | | | |
|---|---|---|---|---|
| | | | | and limited willingness to pay despite clear demographic need. |

Supplementary Table S3. Ex post interpretive alignment of the four-domain analytical framework with NASSS and CFIR

| Domain in the present study | Determinants identified in the cross-case synthesis | Corresponding NASSS domains used for ex post interpretation | Corresponding CFIR domains/constructs used for ex post interpretation | Interpretive role in the present study |
|---|---|---|---|---|
| Technology | Interoperability with existing systems; technological stability and reliability; data security and privacy; usability and interface ergonomics; perceived clinical value | Technology; value proposition; embedding and adaptation over time | Intervention characteristics, including complexity, adaptability, design quality and packaging, and evidence strength and quality; inner setting compatibility where relevant | Supported interpretation of how technical functionality, usability, reliability, perceived value, and fit with existing infrastructures shaped implementation feasibility and adoption. |
| Organization | Leadership, administrative governance, and implementation coordination; Alignment with existing workflows; Organizational infrastructural readiness; Availability of technical support; Interdepartmental and team coordination | Organization; embedding and adaptation over time | Inner setting, including implementation climate, readiness for implementation, leadership engagement, available resources, compatibility, and networks and communication; process, including planning and engaging | Supported interpretation of implementation as an organizational process requiring leadership, workflow integration, infrastructure, technical support, and coordination between teams and units. |
| User | Digital competencies of healthcare staff and other | Adopter system; value proposition; | Characteristics of individuals, including | Supported interpretation of the role of healthcare |

| | | | | |
|---|---|---|---|---|
| | users; structured user training and onboarding; acceptance of technology by professional and patient/end users; engagement of clinicians, frontline personnel, and end users; operational workload implications for staff and care teams | embedding and adaptation over time | knowledge and beliefs about the intervention and self-efficacy; process, including engaging; intervention characteristics, particularly complexity | professionals and other users in adoption, acceptance, skill development, training needs, engagement, and workload implications. |
| System | Funding sustainability; regulatory and legal compliance; institutional and policy-level support; cross-sectoral integration of care; health system readiness, equity, and digital inclusion | Wider institutional and societal context; organization; embedding and adaptation over time | Outer setting, including external policies and incentives, patient needs and resources, and cosmopolitanism; inner setting and process where system-level implementation support shaped local adoption | Supported interpretation of implementation as dependent on broader health system conditions, including funding, regulation, policy support, cross-sector coordination, and system readiness for scaling and sustainability. |

Note. NASSS and CFIR were not used as a priori coding matrices. The alignment shown in this table was conducted only after the empirical determinant structure had been developed through inductive descriptive coding and cross-case synthesis. The purpose of the mapping was to support interpretation and theoretical positioning of the findings, rather than to generate or validate the four-domain framework deductively.
